# Breeding in the City: Non-Linear Associations of Urbanization, Tree Cover, and Human Disturbance with Eurasian Tree Sparrows (*Passer montanus*) Breeding Performance

**DOI:** 10.3390/biology15191746

**Published:** 2026-10-02

**Authors:** Igor Irazusta-Peco, Beatriz Martínez-Miranzo, José I. Aguirre, Eva Banda

**Affiliations:** 1 Biodiversity Monitoring Research Group, Department of Biodiversity, Ecology and Evolution, Faculty of Biological Sciences, Complutense University of Madrid, Jose Antonio Novais, 12, 28040 Madrid, Spain; bmmiranzo@enara.org (B.M.-M.); jaguirre@ucm.es (J.I.A.); ebanda@ucm.es (E.B.); 2 ENARA E.A. S.L., 28290 Las Rozas, Madrid, Spain

**Keywords:** urbanization gradient, tree cover, building height, nest-box monitoring, breeding productivity

## Abstract

Urbanization can alter habitat structure and disturbance regimes, with consequences for bird reproduction. We used 13 years of nest-box monitoring data from 15 urban green spaces in Madrid, Spain, with an average of 866 nest boxes monitored annually, to assess how urbanization, tree cover, building height, road infrastructure, noise, and pedestrian intensity were associated with Eurasian Tree Sparrow (*Passer montanus*) breeding performance. Nest-box occupancy, eggs and hatched chicks per box, nestlings reaching ringing age, and mean number of clutches showed non-linear associations with urbanization, with the highest values generally recorded in partially urbanized areas. Occupancy was 66.9% in these areas, compared with 17.5% in urbanized areas. Tree cover was positively associated with productivity in highly urbanized areas, suggesting that vegetation may be particularly relevant to productivity under highly urbanized conditions. In contrast, greater building height was associated with lower occupancy and reproductive performance. Pedestrian intensity was negatively related to nest-box use and reproductive output, whereas road infrastructure and noise mainly affected later reproductive stages. Overall, breeding performance was associated with the combined characteristics of vegetation, urban structure, and human disturbance. Maintaining partially urbanized green spaces and increasing tree cover in densely built areas may be associated with more favourable breeding conditions for Eurasian Tree Sparrows.

## Simple Summary

Urban development changes the amount of vegetation, the structure of buildings, traffic, noise, and human activity experienced by birds. We used 13 years of nest-box monitoring data from urban green spaces across Madrid (Spain) to examine how these urban characteristics were associated with the reproduction of Eurasian Tree Sparrows. Nest-box use and reproductive output were generally highest in partially urbanized areas, where vegetation and moderate urban development occurred together. Higher tree cover was associated with greater productivity in highly urbanized areas, whereas taller buildings and greater pedestrian activity were associated with poorer breeding conditions. Major roads and noise also affected some stages of reproduction. These findings show that the value of urban green spaces for birds depends not only on their size or amount of vegetation, but also on the surrounding buildings, transport infrastructure, and levels of human disturbance. Maintaining partially urbanized areas, increasing tree cover in densely built neighbourhoods, and placing nest boxes away from heavily disturbed locations may be associated with more favourable breeding conditions for Eurasian Tree Sparrows and other urban cavity-nesting birds.

## 1. Introduction

Urbanization is a global process characterized by the continuous expansion and development of urban areas and represents one of the most extensive and persistent anthropogenic transformations of natural habitats [1,2,3]. Rapid urban growth and the human activities associated with it contribute to habitat fragmentation, loss, and degradation, while also altering the behaviour and life-history traits of wildlife [4,5,6].

Within this context, birds are widely regarded as valuable bioindicators of environmental quality because of their sensitivity to environmental change and the broad range of habitats and trophic levels they occupy [7]. Urbanization generally reduces species richness and modifies the structure of bird communities [8,9]. Accordingly, the degree of urbanization has been widely used to characterize the pressures applied by urban development on bird populations [1,6,10,11,12,13].

Increasing urbanization is generally associated with major transformations in habitat structure, including higher building density and height, reduced vegetation cover, more intense human activity, and increased noise levels [14]. Together, these changes create complex and spatially heterogeneous environments that can influence the demography and reproductive performance of urban birds [15]. Understanding how these environmental factors are associated with reproduction is therefore essential for assessing their consequences for urban bird populations and communities.

Tree cover is one of the main components of urban habitat structure. It influences resource availability [16], provides refuge from predators [17], and is particularly important for species that depend on trees for nesting or foraging [6,18]. Declines in bird abundance have been reported in areas with lower tree cover [16]. Vegetation also contributes to regulating urban microclimates by intercepting solar radiation and promoting evaporative cooling, thereby reducing the intensity of the urban heat island effect [14].

Building height may also influence urban environmental conditions. Tall buildings modify shading patterns, temperature, and air circulation within cities [19,20], potentially affecting the phenology and availability of insect prey. Building height has also been negatively associated with the abundance and distribution of urban birds across seasons and spatial scales [1]. In addition, collision mortality increases in areas dominated by tall buildings [21], representing an additional pressure on urban bird populations.

Other urban infrastructures, particularly roads, are major drivers of habitat loss and fragmentation [22,23]. Roads can affect movement, alter trophic interactions, and increase mortality through vehicle collisions [24,25,26]. They are also important sources of environmental noise [27,28]. Noise pollution has been associated with behavioral and physiological changes in urban wildlife and with reduced reproductive performance in some bird species, including decreases in the number of nestlings and fledglings under prolonged exposure to traffic noise [29].

Human activity represents another major source of disturbance in urban environments [30]. The presence and movement of people can modify how breeding birds and their predators use urban habitats [31]. In particular, high pedestrian density may negatively affect the abundance and distribution of urban birds [1], although its effects may vary among species and stages of the reproductive cycle.

Cavity-nesting species may be especially sensitive to these changes because natural nesting sites, such as tree cavities and abandoned woodpecker holes, are often scarce in urban environments. In addition, competition for available cavities may also affect access to nesting sites, as documented between introduced Ring-necked Parakeets (*Psittacula krameri*) and native cavity-nesting birds [32]. As a result, some species increasingly rely on artificial nesting structures [6]. The Eurasian Tree Sparrow (*Passer montanus* Linnaeus, 1758) can breed in a wide range of artificial cavities, including buildings and nest boxes [33]. Although historically associated with humid and rural environments, the species expanded into European urban areas during the twentieth century, partly in response to the loss and transformation of its original habitats [11]. Despite this expansion, populations have declined in several European regions during recent decades [11,34], particularly in agricultural landscapes affected by intensification, where the availability of winter food resources may be important for breeding populations [35]. Urban environments may therefore provide important breeding habitats for declining farmland birds, including the Eurasian Tree Sparrow [36].

Nest boxes are widely used to compensate for the scarcity of natural cavities in urban environments [29,37]. In addition to their conservation value, they provide a standardized method for monitoring occupancy and reproductive performance across locations and years [38]. Long-term nest-box monitoring can therefore help identify the environmental factors associated with variation in reproductive performance along urban gradients.

In this study, we considered multiple components of the urban environment simultaneously in relation to Eurasian Tree Sparrow breeding performance. We used data from a long-term nest-box monitoring programme to assess the associations of urban landscape structure and human disturbance with the reproductive parameters of the Eurasian Tree Sparrow across an urbanization gradient in Madrid, Spain. Specifically, we examined the associations of urbanization, tree cover, building height, road infrastructure, noise, and pedestrian intensity on nest-box occupancy and reproductive performance. We hypothesized that increasing urbanization and human disturbance would be associated with lower occupancy and reproductive output, whereas greater vegetation cover would weaken these negative relationships. By identifying the urban characteristics associated with breeding performance, this study aims to provide evidence that can inform the planning and management of more bird-friendly urban landscapes.

## 2. Materials and Methods

### 2.1. Study Area

**Figure 1:**
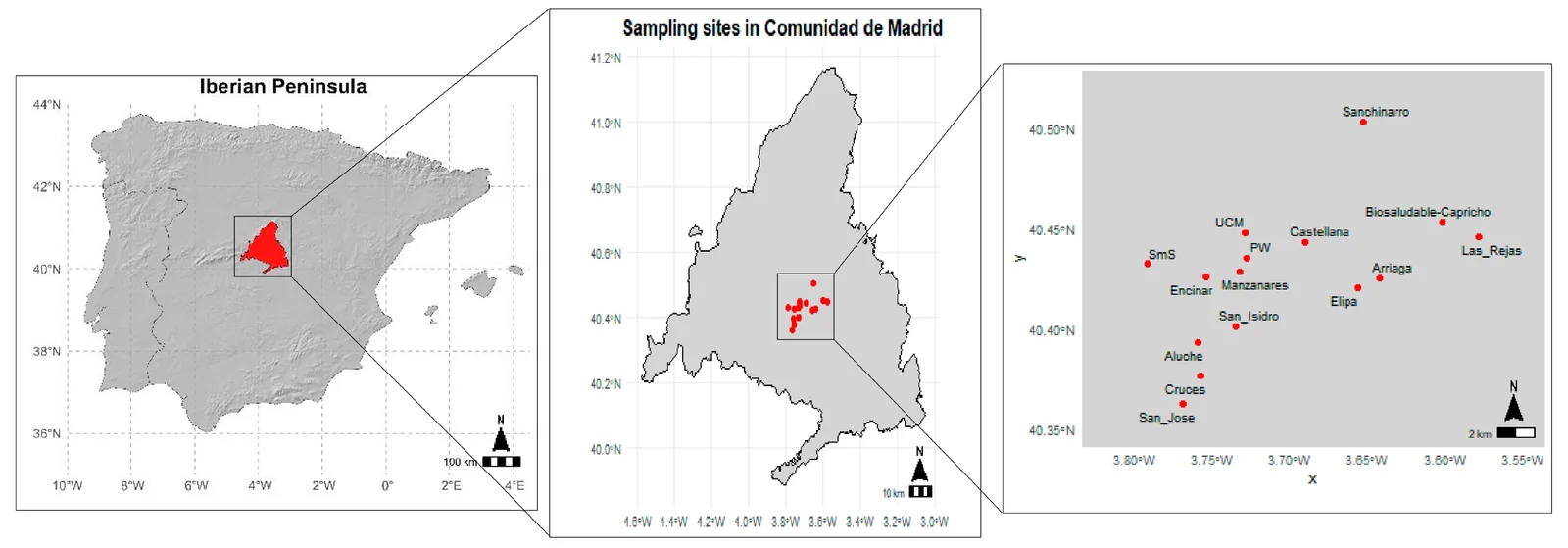
Location of the study areas within the community of Madrid, Spain.

The study was conducted in urban and peri-urban green spaces across Madrid, central Spain, over 13 breeding seasons, from 2012 to 2024. The study locations covered a broad urbanization gradient, ranging from peripheral areas with limited public access and little surrounding built infrastructure to centrally located urban green spaces embedded within densely developed urban areas (Figure 1).

All study locations formed part of a long-term nest-box monitoring programme. However, the temporal coverage differed among locations: two sites were monitored throughout the entire 13-year period, whereas the remaining sites were incorporated progressively between 2019 and 2023. Consequently, the number of locations and nest boxes monitored varied among breeding seasons (Table 1).

### 2.2. Nest-Box Monitoring and Reproductive Variables

Standardized wooden nest boxes (Garden Birds Distribuciones, Montblanc, Spain) measuring 220 mm × 140 mm × 140 mm, with a 28 mm diameter entrance, were used throughout the study. The annual number of monitored nest boxes varied because of differences in the number of active study locations and occasional losses caused by vandalism, theft, or deterioration.

Nest boxes were inspected at least once per week from April to August, with a minimum of sixteen visits to each study location per breeding season. During each inspection, we recorded nest occupancy, clutch initiation, number of eggs, number of hatched chicks, and number of nestlings reaching ringing age. Nestlings were individually marked with uniquely numbered metal rings once they were at least seven days old [39]. Nestlings reaching ringing age were subsequently used as a proxy for fledglings because nestlings surviving to this age were assumed to have a high probability of leaving the nest successfully.

The following reproductive variables were calculated for each combination of location and year: **Occupancy rate**—proportion of available nest boxes occupied by Eurasian Tree Sparrows. **Eggs per nest box**—total number of eggs recorded during the breeding season divided by the number of available nest boxes. **Chicks per nest box**—total number of chicks hatched during the breeding season divided by the number of available nest boxes. **Nestlings reaching ringing age per nest box**—total number of nestlings reaching ringing age divided by the number of available nest boxes. **Mean number of clutches**—mean number of breeding attempts recorded in nest boxes in which egg laying occurred. **Reproductive success**—number of nestlings reaching ringing age divided by the total number of eggs laid. **Productivity**—number of nestlings reaching ringing age divided by the number of nest boxes in which at least one egg was laid. Eggs, chicks, and nestlings reaching ringing age per available nest box therefore integrate nest-box use and subsequent reproductive output, whereas productivity and reproductive success describe breeding performance conditional on reproductive activity.

Standardizing reproductive counts by the number of available or reproductively active nest boxes allowed for comparisons among locations and years with different numbers and densities of nest boxes.

### 2.3. Spatial Extent of the Urban Landscape Analyses

Breeding Eurasian Tree Sparrows generally forage within approximately 300 m of their nests during chick provisioning [40]. To characterize the urban environment surrounding the monitored population, the geographic coordinates of the nest boxes present at each location and in each year were used to construct a minimum convex polygon with the native Convex Hull tool in QGIS 3.28.15 [41]. A 500-m buffer was then added around each polygon to conservatively encompass the typical foraging range of breeding individuals and the wider urban landscape potentially relevant to reproductive performance. For raster-based variables, zonal statistics were used to extract the mean value within each study buffer, whereas vector-based variables were quantified based on the spatial features intersecting each buffer.

The size and shape of these polygons could vary among years because the number and distribution of active nest boxes occasionally changed. Static landscape variables were extracted using the largest spatial extent recorded for each location, whereas temporally varying variables were extracted using the polygon corresponding to each monitoring year or the temporally closest available spatial layer.

### 2.4. Urban Environmental Variables

We considered two groups of explanatory variables. Static variables represented landscape characteristics for which no substantial interannual variation was expected during the study period: urbanization, tree cover, mean building height, and road infrastructure. Dynamic variables represented environmental conditions that could vary among years: noise level and pedestrian intensity. Nest-box density was also included to account for differences in the availability of artificial nesting sites among locations.

The source, spatial format, temporal coverage, and classification of each explanatory variable are summarized in Table 2.

### 2.5. Urbanization Index

Land-cover information was obtained from the Copernicus Urban Atlas 2018. Of the 27 land-cover classes available in the original layer, 19 occurred within the study areas. These were grouped into six ecologically interpretable categories: *green urban areas*, *high-density urban fabric*, *medium-density urban fabric*, *low-density urban fabric*, *industrial*, *commercial*, *public*, *military*, *and private units*, and *other land-cover types*.

The *other land-cover types* category combined 14 land-cover classes that collectively represented less than 10% of the total study area, with no individual class accounting for more than 3%. The proportion of each buffer occupied by each of the six land-cover categories was calculated and used in a principal component analysis to reduce the dimensionality of the land-cover data.

The first principal component, PC1, explained 35.8% of the total variance and was interpreted as an urbanization gradient (eigenvalue = 2.1503; Figure S1 and Table S1). Positive scores were associated with a greater representation of urban green areas and less urbanized environments, whereas negative scores were associated with high- and medium-density urban fabric.

To facilitate interpretation and the representation of potentially non-linear responses, PC1 scores were classified into four urbanization categories: **low urbanized** (PC1 > 1); **partially urbanized** (0 < PC1 ≤ 1); **moderately urbanized** (−1 < PC1 ≤ 0); **urbanized** (PC1 ≤ −1). These cut-off values were used as operational categories to facilitate interpretation and graphical representation of the PCA gradients and do not represent established ecological thresholds.

### 2.6. Tree Cover and Building Height

Tree cover was obtained from the Copernicus Urban Street Tree Layer 2018 and calculated as the proportion of each study buffer occupied by tree canopy. Although tree cover was correlated with the urbanization index (r = 0.77), both variables were retained because they represented biologically distinct attributes of the urban landscape. The urbanization index described the overall composition of the surrounding landscape, whereas tree cover provided a more specific measure of vegetation structure in the vicinity of the nest boxes.

Mean building height was obtained from the Copernicus Urban Atlas Building Height 2012 raster. Mean pixel values were extracted within each study buffer using zonal statistics.

### 2.7. Road Infrastructure

Road data were obtained from the 2024 transport network of the Instituto Geográfico Nacional of Spain. The original road types were consolidated into five categories with comparable structural and functional characteristics. Motorways were grouped with toll motorways, whereas paths were grouped with tracks. The remaining categories were *conventional roads*, *cycle lanes*, and *urban roads*.

The total length of each road category within each study buffer was calculated. These values were included in a principal component analysis to derive a synthetic descriptor of road infrastructure. PC2 was selected because it represented a gradient associated with road type and expected traffic speed, explaining 23.3% of the total variance (eigenvalue = 1.1665; Figure S2 and Table S2). Positive PC2 scores were associated primarily with conventional roads and motorways, whereas negative scores were associated with urban and lower-speed roads.

PC2 was subsequently divided into four categories using the same numerical thresholds applied to the urbanization index: **low-speed roads** (PC2 > 1); **intermediate–low-speed roads** (0 < PC2 ≤ 1); **intermediate–high-speed roads** (−1 < PC2 ≤ 0); and **high-speed roads** (PC2 ≤ −1).

### 2.8. Noise Level

Environmental noise was represented by the day–evening–night noise index (Lden) expressed in decibels. Road-traffic noise data were obtained from the 2016 and 2021 Strategic Noise Maps available through the Madrid Geoportal. The 2016 Lden layer was used for reproductive data from 2012 to 2018, whereas the 2021 Lden layer was used for data from 2019 onwards. This breakpoint was selected because it coincided with the implementation of updated municipal noise-management measures, making the 2021 layer more representative of the environmental conditions during the later study period.

Where spatially explicit Lden data were unavailable, monthly measurements from the municipal noise-monitoring network (LAeq) were used to estimate noise levels. Measurements corresponding to the breeding period, from March to August, were selected and interpolated using inverse distance weighting with a power parameter of 2 [42]. Mean noise levels within each study buffer were subsequently extracted using zonal statistics.

### 2.9. Pedestrian Intensity

Pedestrian intensity was estimated from annual pedestrian-count data available for Madrid between 2019 and 2024. Spatially continuous surfaces were generated by inverse distance weighting, and mean pedestrian intensity was extracted for each study buffer using zonal statistics.

For study locations where municipal pedestrian-count data were unavailable, including the Somosaguas campus, annual information on enrolled students, teaching and research staff, and administrative and services staff was obtained from the Institutional Intelligence Centre of the Complutense University of Madrid. These values were divided by the corresponding study area to obtain an estimate of pedestrian density.

Only information corresponding to the breeding period from March to August was incorporated into the analyses. Spatial layers derived from different sources were harmonized to ensure consistent alignment and spatial resolution before variable extraction.

### 2.10. Nest-Box Density

Nest-box density was calculated for each location and year as the number of available nest boxes per hectare of the area occupied by the nest-box array. This variable was included to control differences in the local availability and spatial concentration of artificial nesting sites.

### 2.11. Statistical Analyses

Principal component analyses were used to derive the urbanization and road-infrastructure indices described above. A correlation matrix was also constructed to evaluate associations among the explanatory variables before model fitting (Figure S3). The results showed that tree cover was strongly associated with habitat composition and the urbanization gradient, while mean building height was strongly associated with noise level; however, correlations varied substantially among predictors.

Generalized linear models were used to examine the relationships between urban environmental variables and each reproductive response. Occupancy rate and reproductive success, both expressed as proportions between 0 and 1, were analysed using models with a quasibinomial error distribution and a logit link. Because Akaike’s information criterion cannot be calculated directly for quasibinomial models, candidate models were initially fitted using a binomial distribution for model comparison and were subsequently refitted using a quasibinomial distribution for coefficient estimation and statistical inference.

The remaining response variables (eggs, chicks, and nestlings reaching ringing age) per nest box, productivity, and mean number of clutches) were analysed using Gaussian error distributions. The initial candidate models included urbanization category, tree cover, mean building height, road category, noise level, pedestrian intensity, nest-box density, and year when appropriate. The urbanization x tree-cover interaction was evaluated for all reproductive response variables and was retained only for productivity, where its inclusion improved model fit, with continuous predictors retained in their original units to facilitate direct ecological interpretation of the model estimates.

To assess whether repeated sampling among locations and years introduced additional hierarchical variation, we also fitted alternative mixed-effects models including location and year as random intercepts. Variance estimates for both random effects were negligible, and models including both terms resulted in singular fits. We therefore retained the GLM framework for the final analyses. Individual nest boxes were not treated as independent analytical units because predictors and response variables were summarized at the location-year level.

The initial predictor set was defined a priori from the ecological hypotheses of the study. Model selection was conducted using a stepwise procedure based on Akaike’s information criterion (AIC) [43] as a model-reduction procedure to identify the most parsimonious subset of predictors for each reproductive response. The model with the lowest AIC was considered the most parsimonious representation of the data. We acknowledge that stepwise procedures may produce model-selection uncertainty, particularly in observational datasets with correlated predictors, and selected effects were therefore interpreted cautiously. Model residuals and assumptions were examined for the selected models, and correlations among predictors were considered when interpreting parameter estimates, while multicollinearity was assessed using VIF and adjusted GVIF values (Table S3).

As an additional sensitivity analysis specifically addressing temporal trends, we restricted the dataset to Parque del Oeste (PW) and Campus Moncloa (UCM), the two locations monitored throughout 2012–2024, and fitted Gaussian models including year, location, and their interaction (year x location) for eggs, chicks, and nestlings reaching ringing age per nest box.

All analyses were performed in R version 4.4.2 [44]. Stepwise model selection was conducted using the MASS package version 7.3.61, and analysis-of-variance tables for the selected models were obtained using the car package version 3.1.3. Statistical significance was assessed at α = 0.05.

## 3. Results

Urbanization category was consistently associated with nest-box occupancy and reproductive output. Occupancy, eggs per nest box, chicks per nest box, nestlings reaching ringing age, and mean number of clutches were all highest in partially urbanized areas. Building height and pedestrian intensity generally showed negative associations with breeding activity, whereas the associations with tree cover, roads, and noise were concentrated in specific reproductive parameters.

### 3.1. Breeding Activity Along the Urbanization Gradient

**Figure 2:**
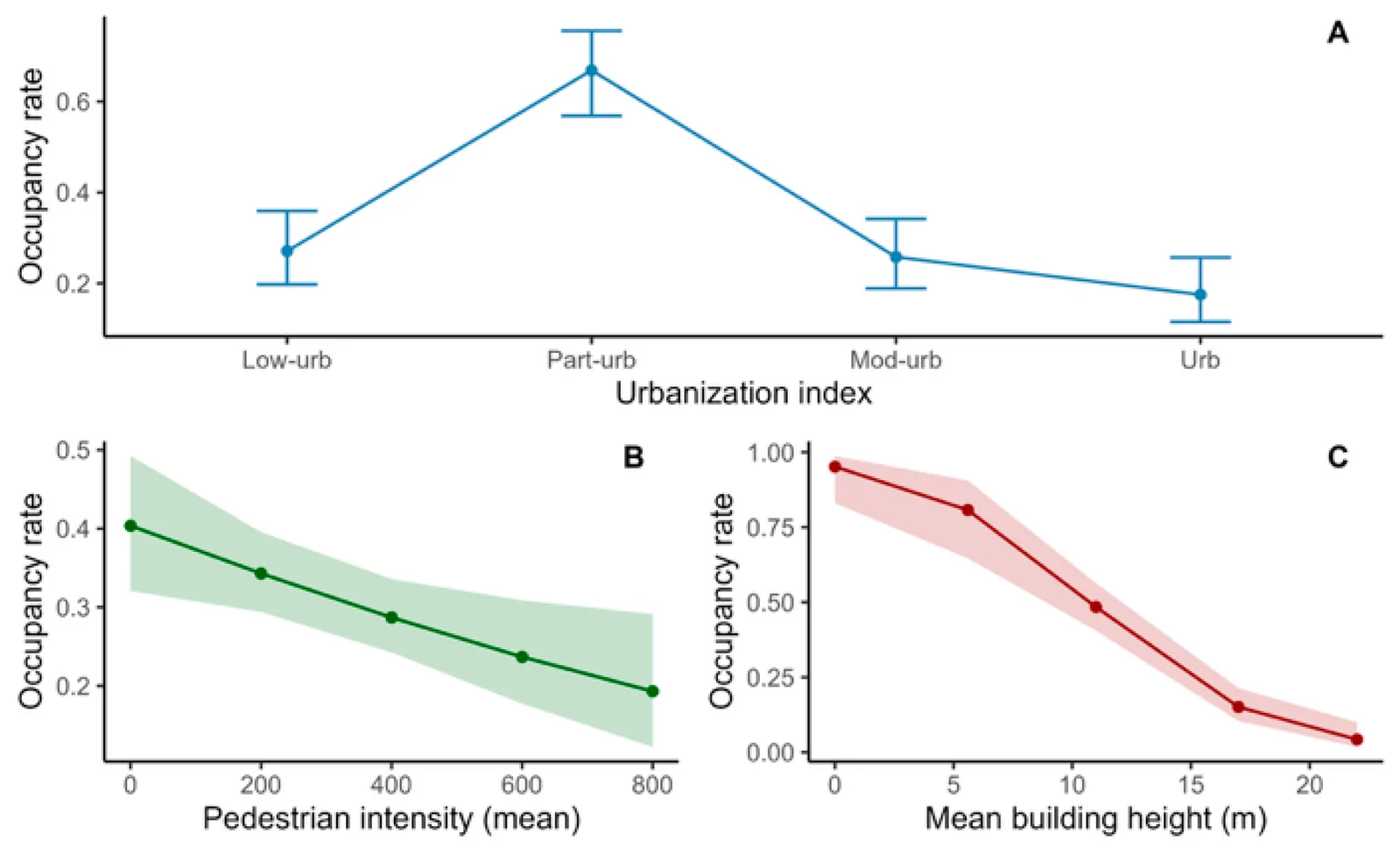
Associations of urbanization category (**A**), pedestrian intensity (**B**), and mean building height (**C**) with Eurasian Tree Sparrow nest-box occupancy. Lines or points represent model predictions, and shaded areas or error bars represent 95% confidence intervals.

Nest-box occupancy was associated with urbanization category, pedestrian intensity, and mean building height (AIC = 58.26; Figure 2). Occupancy was higher in partially urbanized than in low urbanized areas (β = 1.692 ± 0.300, *p* < 0.001). In contrast, occupancy decreased with increasing pedestrian intensity (β = −0.0013 ± 0.0005, *p* = 0.012) and mean building height (β = −0.277 ± 0.052, *p* < 0.001). Occupancy did not differ significantly between low urbanized areas and either moderately urbanized or urbanized areas.

The number of eggs per nest box showed a similar pattern (AIC = 207.44). Partially urbanized areas had more eggs per nest box than low urbanized areas (β = 3.249 ± 0.816, *p* < 0.001), whereas pedestrian intensity was negatively associated with this response (β = −0.009 ± 0.001, *p* < 0.001). Neither moderately urbanized nor urbanized areas differed significantly from low urbanized areas (Figure 2).

**Figure 3:**
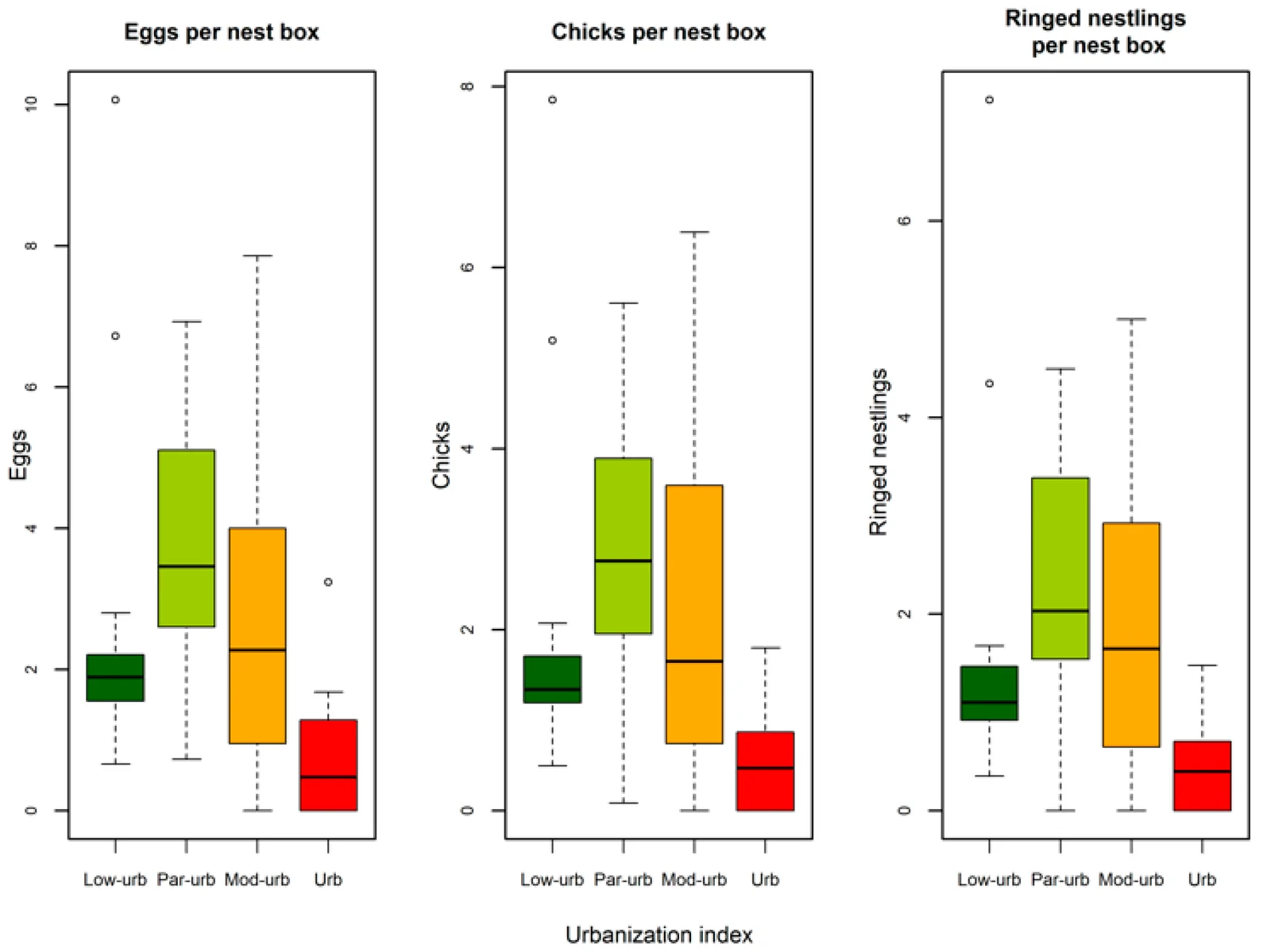
Number of eggs, hatched chicks, and nestlings reaching ringing age per nest box across the urbanization categories. Boxplots show the observed distributions within each urbanization category.

The number of chicks hatched per nest box was also higher in partially urbanized areas (β = 2.390 ± 0.701, *p* = 0.002; AIC = 189.16) and declined with increasing pedestrian intensity (β = −0.007 ± 0.001, *p* < 0.001). No significant differences were detected between low urbanized areas and the two more urbanized categories (Figure 3).

The number of nestlings reaching ringing age per nest box followed the same general urbanization pattern (Figure 3). Values were higher in partially urbanized areas than in low urbanized areas (β = 2.506 ± 0.602, *p* < 0.001), whereas no significant differences were found for moderately urbanized or urbanized areas. Pedestrian intensity remained negatively associated with this later reproductive stage, although the estimated effect was weaker than for eggs and hatched chicks (β = −0.001 ± 0.001, *p* = 0.039).

### 3.2. Productivity and Reproductive Success

Productivity was associated with urbanization category, tree cover, mean building height, and the interaction between urbanization category and tree cover. Including the interaction improved model fit from AIC = 248.62 to AIC = 238.19.

**Figure 4:**
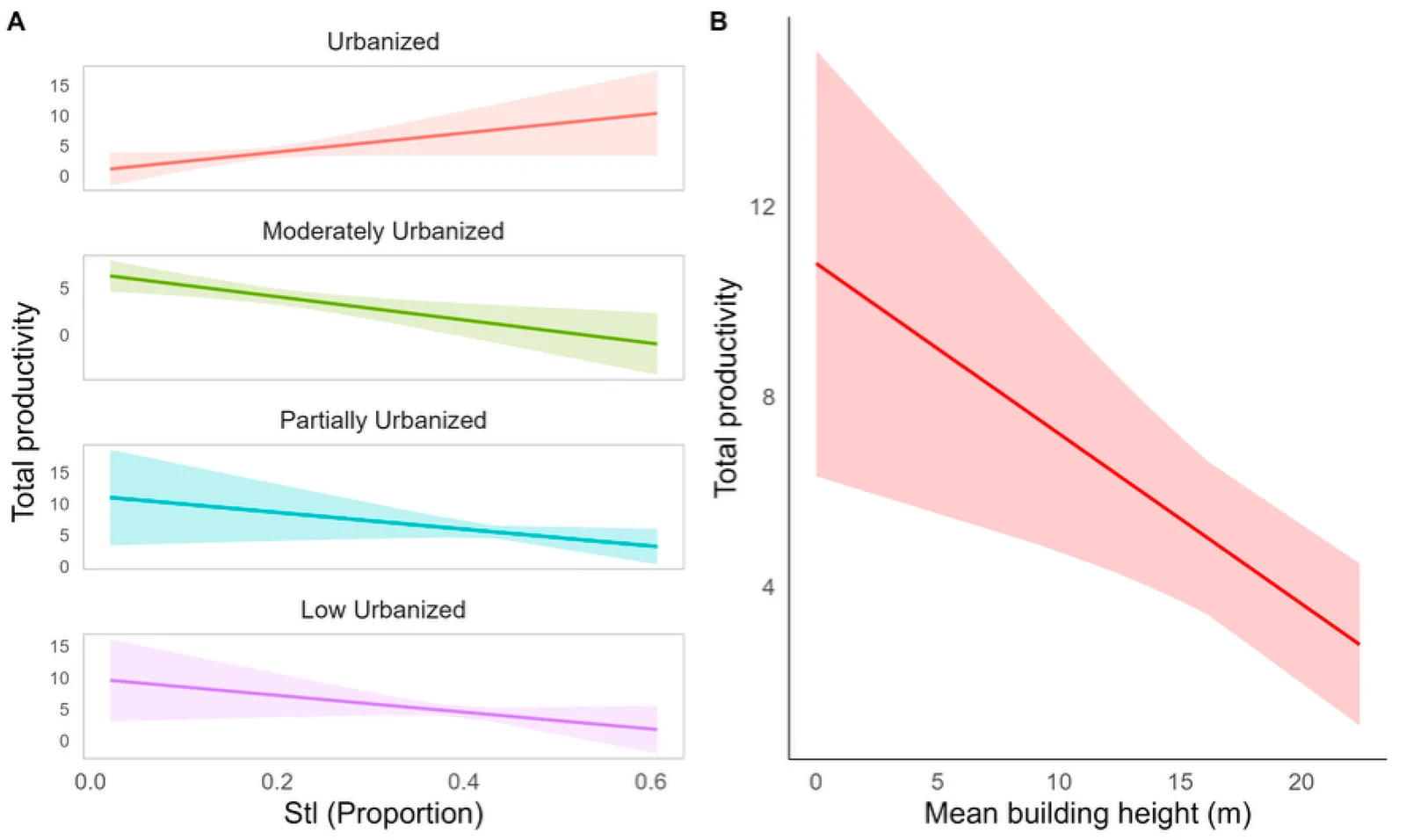
Associations of the interaction between urbanization category and tree cover (Stl) on productivity (**A**), and the relationship between mean building height and productivity (**B**). Predictions are shown with shaded areas representing 95% confidence intervals. Confidence intervals widen for combinations of urbanization category and tree cover that were poorly represented in the observed data.

In urbanized areas, productivity was positively associated with tree cover, as indicated by the positive interaction between the urbanized category and tree cover (β = 29.230 ± 8.985, *p* = 0.002; Figure 4A). In the absence of this interaction, urbanized areas had lower predicted productivity than low urbanized areas (β = −9.103 ± 3.060, *p* = 0.004). However, the positive interaction indicates that this difference became smaller at higher levels of tree cover.

The interaction between tree cover and moderately urbanized areas was not significant (β = 1.017 ± 9.251, *p* = 0.913). The interaction for partially urbanized areas could not be estimated because of the limited variation in tree cover within this category. The overall main effect of tree cover was not significant, indicating that its association with productivity depended on urbanization context rather than being consistent across all study areas.

Productivity also declined with increasing mean building height (β = −0.357 ± 0.112, *p* = 0.002; Figure 4B).

Mean building height was the only predictor significantly associated with reproductive success. Reproductive success decreased as building height increased (β = −0.209 ± 0.067, *p* = 0.003). No significant associations were detected for nest-box density, pedestrian intensity, noise level, or tree cover.

### 3.3. Roads, Noise, and Breeding Attempts

Road category and noise level were mainly associated with later reproductive stages. The number of nestlings reaching ringing age was lower in areas characterized by intermediate–high-speed roads (β = −1.825 ± 0.551, *p* = 0.002) and high-speed roads (β = −2.008 ± 0.696, *p* = 0.006) than in areas dominated by low-speed roads. No significant difference was detected for intermediate–low-speed roads.

The number of nestlings reaching ringing age also decreased as the mean noise level increased (β = −0.137 ± 0.022, *p* < 0.001). Neither road category nor noise level was retained as a significant predictor of the earlier reproductive responses.

**Figure 5:**
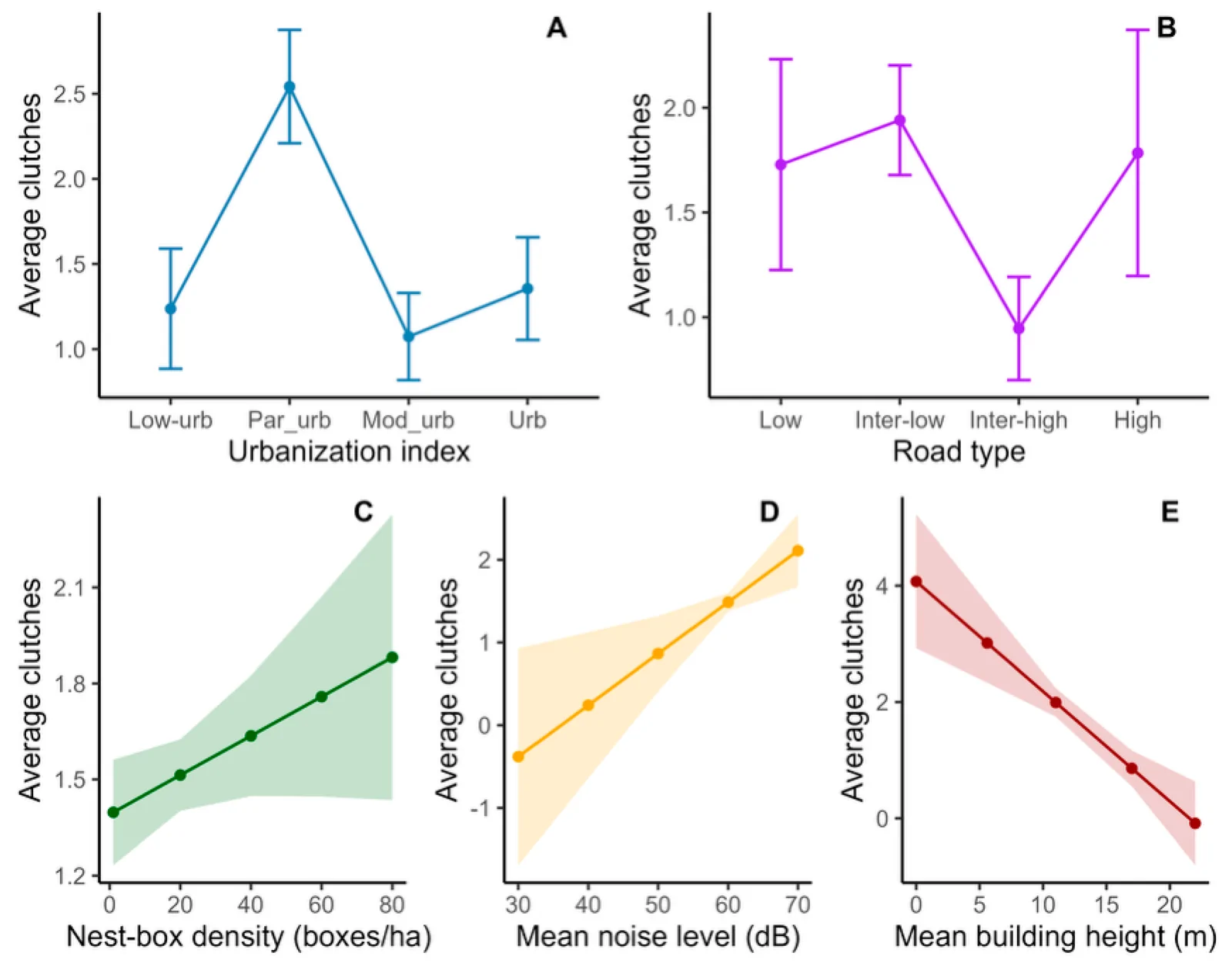
Modelled relationships between mean clutch number and urbanization category (**A**), road category (**B**), nest-box density (**C**), mean noise level (**D**), and mean building height (**E**). Predictions are shown with shaded areas representing 95% confidence intervals.

The mean number of clutches was higher in partially urbanized areas than in low urbanized areas (β = 1.305 ± 0.285, *p* < 0.001; AIC = 79.70; Figure 5A). It declined with increasing building height (β = −0.189 ± 0.042, *p* < 0.001; Figure 5E) and was lower in areas characterized by intermediate–high-speed roads than in those dominated by low-speed roads (β = −0.782 ± 0.290, *p* = 0.010; Figure 5B). Contrary to the general pattern observed for nestlings reaching ringing age, mean clutch number increased with noise level (β = 0.062 ± 0.021, *p* = 0.006; Figure 5D).

Nest-box density showed a positive but non-significant association with mean clutch number (β = 0.006 ± 0.003, *p* = 0.085; Figure 5C).

### 3.4. Temporal Variation

**Figure 6:**
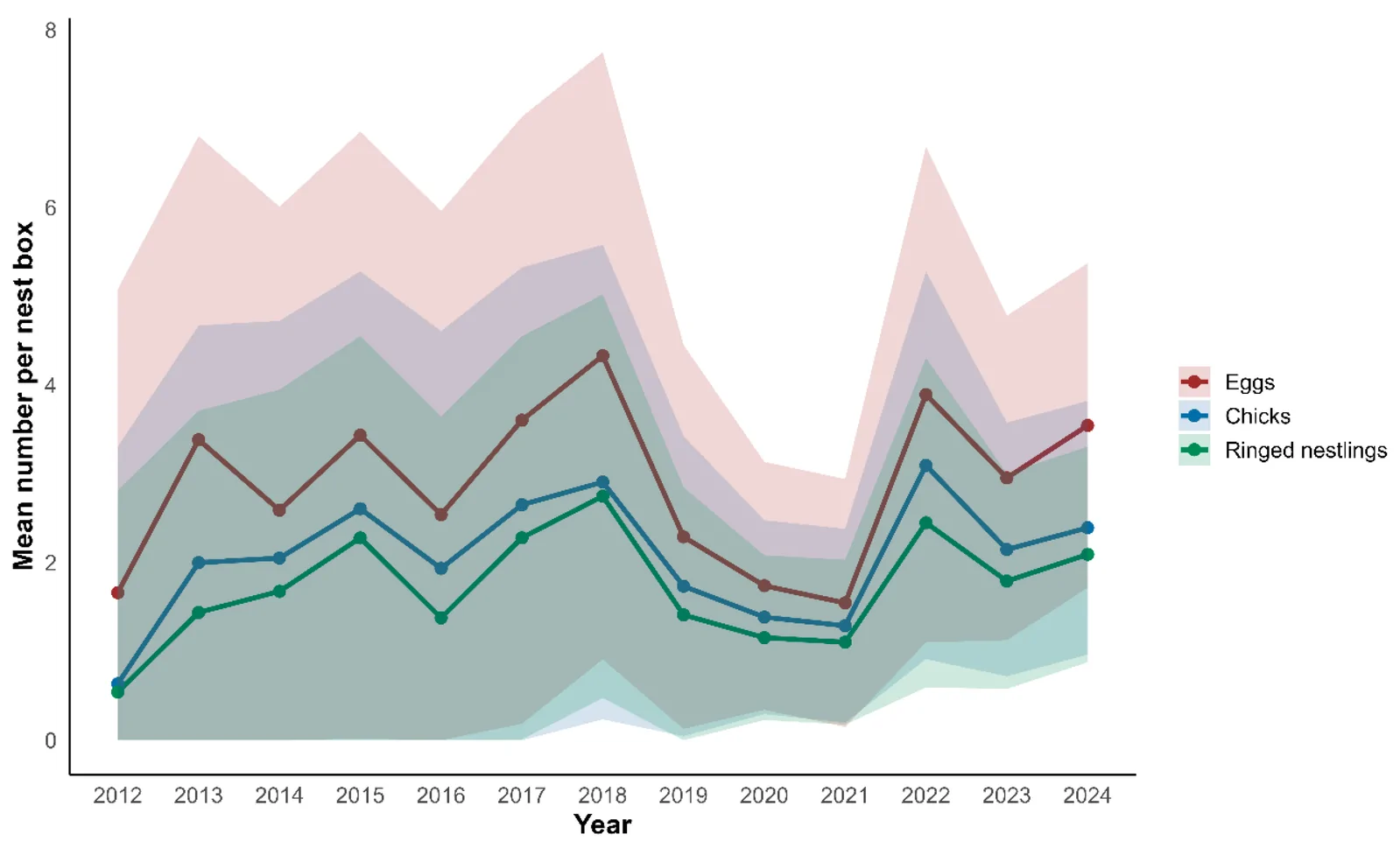
Annual variation in the mean number of eggs, hatched chicks, and nestlings reaching ringing age per nest box between 2012 and 2024. Points represent annual means, lines represent fitted temporal patterns, and shaded areas represent 95% confidence intervals.

Interannual variation was observed in egg and chick numbers, but these patterns should be interpreted descriptively because sampling effort and site composition differed among years (Figure 6). Relative to 2012, egg numbers were higher in 2018, 2021, 2022, 2023, and 2024, with the largest estimated differences occurring during the final three years of the study. Similarly, chick numbers were higher than in 2012 from 2018 onwards, except that the evidence for a difference in some earlier years was weaker.

Despite these increases during the initial stages of reproduction, the number of nestlings reaching ringing age showed no significant temporal trend. Occupancy, productivity, and reproductive success also remained comparatively stable over the study period.

To assess whether the observed temporal patterns could be influenced by the unbalanced monitoring design, we repeated the temporal analysis using only Parque del Oeste (PW) and Campus Moncloa (UCM), the two locations monitored continuously from 2012 to 2024. In these consistently monitored locations, no significant linear temporal trend was detected for eggs, chicks, or nestlings reaching ringing age (*p* = 0.216, 0.581, and 0.599, respectively). Site x year interactions were also non-significant for all three variables (*p* = 0.610, 0.997, and 0.930, respectively).

Full coefficients for the final models are provided in Table S4.

## 4. Discussion

Our results show that the breeding performance of Eurasian Tree Sparrows was associated with several complementary dimensions of the urban environment. Urbanization category, building height, and pedestrian intensity were related to nest-box occupancy and reproductive output, whereas tree cover, road infrastructure, and noise showed more context-dependent effects. The highest values for occupancy, eggs, hatched chicks, nestlings reaching ringing age, and mean number of clutches were generally recorded in partially urbanized areas. This indicates that breeding performance did not respond linearly to urbanization, but instead peaked at intermediate positions along the urban gradient. In addition, tree cover was positively associated with productivity in highly urbanized areas, while greater building height was consistently associated with poorer reproductive performance.

### 4.1. Response to Urbanization

Urbanization category was one of the most consistent predictors of nest-box use and reproductive output. However, the observed pattern cannot be described simply as a progressive decline along the urbanization gradient. Partially urbanized areas showed higher occupancy and reproductive values than the least urbanized areas, whereas differences between the least and more urbanized categories were not always statistically significant.

Previous studies have shown that urban insectivorous birds often lay smaller clutches and produce fewer fledglings than populations breeding in less urbanized environments [45,46]. Nevertheless, responses may be non-linear because intermediate urban environments can combine resources derived from human activity with vegetation and other habitat elements characteristic of more natural areas [45].

The Eurasian Tree Sparrow can exploit urban environments and use artificial nesting structures while still depending on surrounding habitats for food and shelter [11,33]. Partially urbanized areas may therefore provide a favourable combination of nesting opportunities, vegetation, open foraging areas, and supplementary anthropogenic resources. Similar responses have been described in urban bird communities, where habitat heterogeneity and moderate levels of urban development may support greater resource diversity than either highly built environments or areas with lower levels of urbanization [47].

This pattern suggests that the ecological quality of an urban green space cannot be inferred solely from its position along a simple urban–natural gradient. Instead, breeding suitability may depend on the particular combination and spatial arrangement of built structures, vegetation, disturbance, and food resources. From a planning perspective, maintaining transitional and partially urbanized habitats may therefore be as important as preserving the least urbanized green spaces.

### 4.2. Tree Cover as a Context-Dependent Buffer

Tree cover did not show a uniform positive association with all reproductive parameters. Instead, its effect was concentrated on productivity and depended on urbanization category. In highly urbanized areas, productivity increased as tree cover increased. This pattern is consistent with the potential ecological benefits of urban trees.

Urban trees provide multiple ecological functions, including food resources, shelter, thermal regulation, and structural complexity [48,49]. Greater tree cover has also been associated with increased abundance of insectivorous and omnivorous birds [16], while vegetation can reduce the exposure of nests to visually oriented predators [17]. These processes could contribute to the higher productivity observed in more heavily vegetated urban locations.

However, tree cover was not significantly associated with occupancy, the total numbers of eggs or chicks per available nest box, or reproductive success. Therefore, its effect should not be interpreted as a general enhancement of all stages of reproduction. Rather, the results suggest that tree cover was positively associated with the output of nest boxes in which breeding has already been initiated, particularly where the surrounding urban matrix is highly developed.

The lack of sufficient variation in tree cover within some urbanization categories limits the interpretation of the interaction. In particular, the effect could not be estimated reliably for partially urbanized areas. Consequently, the apparent association between tree cover and productivity should be interpreted primarily for the highly urbanized settings represented in this study. Additional observations covering a wider range of tree-cover values within each urban category would help determine whether this relationship is consistent across the urban landscape.

### 4.3. Building Height and Urban Form

Mean building height was negatively associated with nest-box occupancy, productivity, reproductive success, and mean number of clutches. Together with the urbanization index, it was therefore one of the most consistent predictors of reproductive performance.

Previous research has reported negative associations between building height and the abundance and distribution of Eurasian Tree Sparrows [1]. Tall buildings can alter shading patterns, air circulation, and local temperatures [19,20]. These changes may affect vegetation and arthropod availability, thereby modifying the quality of the foraging environment around nest sites [50,51,52]. Because nest-site selection depends partly on resources available in the surrounding landscape [37], reduced habitat quality near taller buildings could decrease both the probability of nest-box occupation and subsequent reproductive performance.

Building height may also represent a broader indicator of urban form. Areas dominated by tall buildings are often associated with greater built density, reduced ground-level vegetation, stronger shading, and more intense human activity. It is therefore difficult to attribute the observed effects to building height alone. Although the models accounted for several correlated urban variables, building height may capture environmental characteristics not fully represented by the remaining predictors.

### 4.4. Road Infrastructure and Noise

Road category was mainly associated with later reproductive outcomes. The number of nestlings reaching ringing age and the mean number of clutches were lower in areas characterized by intermediate–high-speed or high-speed roads. This pattern suggests that road infrastructure may become particularly relevant after breeding has been initiated.

Roads are major sources of habitat fragmentation and can reduce connectivity between nesting and foraging areas [24,26]. Fragmented landscapes can increase the distance and effort required to obtain food [53,54,55], potentially reducing the resources available for repeated breeding attempts or chick provisioning.

The absence of significant road effects on occupancy, productivity, or reproductive success indicates that the relationship was not uniform across the reproductive cycle. One possibility is that road infrastructure may be associated with cumulative differences in the number of young produced per available nest box, while having less influence on the proportional success of nests in which breeding occurs. Alternatively, unmeasured local habitat conditions may partly compensate for road-related pressures in some nest-box groups.

Noise level also showed contrasting associations among reproductive variables. Higher noise was associated with fewer nestlings reaching ringing age, which is consistent with studies showing that chronic anthropogenic noise can alter parental behaviour, stress responses, and reproductive performance [29,56]. Noise can interfere with communication between adults and young or affect provisioning behaviour, potentially reducing offspring survival.

In contrast, the mean number of clutches increased with noise level. Similar selection of noisy breeding environments has been documented in some passerines, and predator activity or species interactions have been suggested as possible explanations [57,58]. Nevertheless, this positive association should be interpreted cautiously. Noise may be correlated with other unmeasured features of urban areas, and a greater number of breeding attempts does not necessarily imply greater reproductive success. Indeed, the simultaneous negative association between noise and the number of nestlings reaching ringing age could indicate that repeated breeding attempts partly reflect compensation for unsuccessful or less productive earlier attempts, although this possibility could not be tested with the present data.

### 4.5. Pedestrian Disturbance and Nest-Box Use

Pedestrian intensity was negatively associated with occupancy and with the number of eggs, hatched chicks, and nestlings reaching ringing age per available nest box. In contrast, it was not significantly associated with productivity or reproductive success. This distinction is consistent with pedestrian intensity being more strongly related to whether and how often nest boxes were used, rather than the proportional outcome of breeding attempts once they had begun.

Human disturbance can affect birds at the individual, population, and community levels [59]. High pedestrian activity may reduce the attractiveness of nesting sites or increase the frequency of disturbance near occupied boxes [1]. Birds may avoid nest boxes located close to heavily used paths or recreational areas, as documented in other urban settings [60].

The lack of a significant association with productivity or reproductive success may indicate that pairs selecting nest boxes in areas with higher pedestrian intensity are relatively tolerant of human presence. It may also result from differences in the denominators of the reproductive metrics. Variables expressed per available nest box incorporate both nest-site selection and breeding performance, whereas productivity and reproductive success include only boxes in which reproduction was initiated. Consequently, pedestrian intensity may reduce the number of active nests at a location without strongly affecting the outcome of the subset that remains occupied.

Human activity can also modify predator behaviour. In some settings, human presence may discourage certain predators, while in others, it may facilitate access to anthropogenic food or increase the activity of urban-adapted predators such as corvids, cats, and rats [29]. Because predator abundance and nest predation were not measured, these alternatives cannot be distinguished with the present data.

### 4.6. Temporal Patterns and Study Limitations

Egg and chick numbers varied among years, whereas occupancy, productivity, reproductive success, and the number of nestlings reaching ringing age remained comparatively stable. The differences observed among years in the early reproductive stages were not accompanied by corresponding differences in the number of nestlings reaching ringing age.

However, temporal patterns must be interpreted cautiously because the monitoring design was unbalanced. Some locations were surveyed throughout the 13-year study, whereas others were incorporated only during the final years. Apparent annual differences may reflect both temporal variation and changes in the composition of monitored locations and should therefore not be interpreted as city-wide population trends. As a sensitivity analysis, we repeated the temporal analysis using only the two locations monitored continuously from 2012 to 2024 (PW and UCM). No significant linear temporal trends were detected for eggs, chicks, or nestlings reaching ringing age, and site x year interactions were not significant. These results provide no evidence of a consistent long-term temporal trend across the consistently monitored locations. For this reason, the present study provides stronger evidence for spatial associations with urban variables than for city-wide temporal trends.

The observational design also prevents causal attribution. Urban variables were often correlated and may represent broader combinations of landscape structure and human use. Environmental predictors were derived from datasets with different reference years. Land cover, tree cover, building height, and road infrastructure were treated as relatively stable descriptors of landscape structure, whereas noise and pedestrian data were assigned according to their temporal availability. These variables should therefore be interpreted as approximations of environmental conditions rather than exact annual measurements for every breeding season. The sensitivity of the observed associations to alternative buffer sizes was not evaluated. In particular, pedestrian intensity did not directly measure disturbance or distinguish between occasional and permanent footfall. These procedures allowed for the integration of heterogeneous sources across a long-term monitoring program, but they inevitably introduced some methodological heterogeneity and differences in spatial and temporal precision. Because model reduction was based on stepwise AIC, individual coefficient estimates may also be sensitive to the selected model structure. Accordingly, the results are interpreted primarily as associations rather than as evidence of causal relationships. A further limitation concerns the urbanization x tree cover interaction: tree cover showed no variation within the partially urbanized category, preventing estimation of the corresponding interaction term for this category.

Finally, the use of nestlings reaching ringing age as a proxy for fledging represents another limitation. Although reaching ringing age represents an advanced stage of nest development, successful departure from the nest was not directly confirmed in every case. The results should therefore be interpreted as reproductive output up to ringing age rather than as a direct measurement of fledging success.

### 4.7. Implications for Urban Landscape Planning

Despite these limitations, the study identifies several urban landscape characteristics associated with Eurasian Tree Sparrow reproduction. The highest breeding values in partially urbanized areas indicate that intermediate landscapes combining vegetation, open areas, nesting opportunities, and moderate urban development may provide particularly favourable conditions.

The interaction between tree cover and urbanization further suggests that vegetation may be relevant to reproductive conditions even where the surrounding matrix is strongly urbanized. Increasing and maintaining tree cover may therefore be particularly relevant in densely developed neighbourhoods. However, tree planting alone may not compensate fully for other structural pressures, as productivity and reproductive success were also lower around taller buildings.

The negative associations with pedestrian intensity, high-speed roads, and noise indicate that the spatial configuration and use of urban green spaces are also important. Nest boxes and other biodiversity interventions should not be located solely according to the presence of trees or available green space. Their surroundings should also be evaluated in terms of human circulation, road infrastructure, building form, and acoustic conditions.

Urban management aimed at supporting cavity-nesting birds should therefore prioritise heterogeneous and partially urbanized green spaces, retain or increase tree cover within highly urbanized areas, and provide nesting opportunities away from the most intensively used pedestrian corridors and major transport infrastructures. More broadly, the results show that urban biodiversity conservation depends not only on the amount of green space, but also on its structural context, connectivity, and level of disturbance.

Overall, Eurasian Tree Sparrow breeding performance was highest in urban landscapes that combined natural elements with moderate levels of urban development. The findings support an integrated approach to urban planning in which vegetation, built form, transport infrastructure, and human activity are considered jointly rather than as independent components. Such an approach could be relevant to the ecological functioning of urban green spaces and contribute to the design of more bird-friendly cities.

## 5. Conclusions

This study shows that Eurasian Tree Sparrow breeding performance is associated with multiple components of the urban environment, including urbanization level, tree cover, building height, road infrastructure, noise, and pedestrian activity. Reproductive parameters were generally highest in partially urbanized areas, indicating that the species did not respond linearly to the urbanization gradient. Instead, intermediate urban landscapes may provide a favourable combination of vegetation, nesting opportunities, and access to resources.

Tree cover played a particularly important role in highly urbanized areas, where increasing vegetation was associated with higher productivity. In contrast, greater building height was consistently related to lower nest-box occupancy and reproductive performance. Pedestrian intensity was mainly associated with reduced nest-box use and lower reproductive output per available box, whereas road infrastructure and noise primarily affected later reproductive stages.

These findings emphasize that urban bird conservation should consider not only the amount of urban green space, but also its structural characteristics, surrounding built environment, accessibility, and level of disturbance. Maintaining partially urbanized urban green spaces, increasing tree cover in densely urbanized areas, and locating nest boxes away from heavily used pedestrian corridors, tall buildings, and major transport infrastructures may be relevant for supporting the breeding conditions for Eurasian Tree Sparrows.

Long-term nest-box monitoring provides a valuable approach for assessing the responses of cavity-nesting birds to urban environmental change. However, because the study was observational, the reported relationships should be interpreted as associations rather than direct causal effects. Future research combining long-term monitoring with measurements of food availability, microclimate, predation, and actual fledging success would help clarify the mechanisms underlying the observed patterns.

## Figures and Tables

**Table 1:** Study locations, mean number of nest boxes, urbanization category derived from the land-cover principal component analysis, and temporal coverage of the reproductive data.

Location	Mean Number of Nest Boxes	Urbanization Index	Temporal Range (Number of Seasons)
Aluche	60	Urbanized	2020–2021 (2)
Arriaga	40	Moderately urbanized	2020–2021 (2)
Capricho-Biosaludable	32	Urbanized	2020–2024 (5)
Castellana	118	Urbanized	2019–2021 (3)
Cruces	72	Urbanized	2020–2021 (2)
Elipa	20	Urbanized	2023–2024 (2)
Encinar (ESP)	61	Low urbanized	2023–2024 (2)
Las Rejas	37	Moderately urbanized	2020–2021 (2)
Manzanares	25	Moderately urbanized	2019–2021 (3)
Parque del Oeste (PW)	64	Partially urbanized	2012–2024 (13)
San Isidro	20	Moderately urbanized	2023–2024 (2)
San José	34	Moderately urbanized	2020–2021 (2)
Sanchinarro	36	Moderately urbanized	2020–2021 (2)
Somosaguas (SmS)	50	Moderately urbanized	2019–2024 (6)
Campus Moncloa (UCM)	197	Low urbanized	2012–2024 (13)

**Table 2:** Description, source, spatial format, and temporal classification of the urban environmental variables included in the analyses.

Variable	Description	Source	Layer Type (Spatial Resolution)	Variable Type
Urbanization index	Functions assigned to areas within the buffer. PCA and subsequent reclassification of PC1 into four categories	Urban Atlas Land Cover 2018 (Copernico)	Vector (-)	Static
Tree cover (%)	Percentage of the total areas occupied by trees within the buffer, obtained by intersecting the source layer	Urban Street Tree Layer 2018 (Copernico)	Vector (-)	Static
Mean building height (m)	Mean building height obtained through zonal statistics from the source layer	Urban Atlas Building Height 2012 (Copernico)	Raster (5 m)	Static
Road categories (m)	Length of each type of road within the study areas. PCA and subsequent reclassification of PC2 into four categories	Transport Networks 2024 (National Geographic Institute of Spain)	Vector (-)	Static
Noise level (dB)	Noise level per road (Lden) and sound levels at Somosaguas obtained by interpolation of monthly continuous noise pollution data (LAeq)	Strategic Noise Maps 2016 and 2021 Historical monthly noise pollution data (Geoportal Madrid)	Raster (5 m)	Dynamic
Pedestrian intensity	Mean number of pedestrians in the study area, obtained by interpolation of annual counts, combined with student and staff (PDI/PAS) density at Somosaguas.	Annual pedestrian counts, 2019–2024 (Geoportal Madrid); Somosaguas campus data, 2019–2024	Raster (5 m)/-	Dynamic

## Data Availability

The data are not publicly available because they include sensitive information (e.g., precise locations of monitored breeding sites) that could pose risks to the studied populations. Anonymised data may be available from the corresponding author upon reasonable request.

## Supplementary Materials

The following supporting information can be downloaded at: https://www.mdpi.com/article/10.3390/biology15191746/s1, Figure S1: Principal component scores and loadings. The axes represent the first two principal components, and the arrows indicate the contribution and direction of each variable. *hab_highurban* denotes habitats with high urban density, *hab_midurban* medium-density urban areas, *hab_lowurban* low-density urban areas, *hab_other* other habitat types, *hab_green* green areas, and *hab_indcom* commercial and industrial areas; Figure S2: Principal component scores and loadings. The axes represent the first two principal components, and the arrows indicate the contribution and direction of each variable. *autov* denotes motorways and highways, *camino* paths and tracks, *carret* conventional roads, *bici* bike lanes, and *urbano* urban roads; Figure S3: Correlation matrix of the urban variables included in the study. Colors indicate the direction and magnitude of correlations (red: negative; blue: positive), and color intensity reflects the strength of the relationship, with values ranging from −1 to +1; Table S1: Variance explained and loadings of the principal components describing the degree of urbanization; Table S2: Variance explained and loadings of the principal components describing road-related variables; Table S3: Variance inflation factors (VIF), generalized variance inflation factors (GVIF), and adjusted GVIF values for predictors included in the final models. Table S4: Full coefficients for the final models.

## References

[1] Zhang S., Zheng G. (2010). Effect of Urbanization on the Abundance and Distribution of Tree Sparrows (*Passer montanus*) in Beijing. Chin. Birds.

[2] Morelli F., Benedetti Y., Ibáñez-Álamo J.D., Jokimäki J., Mänd R., Tryjanowski P., Møller A.P. (2016). Evidence of Evolutionary Homogenization of Bird Communities in Urban Environments across Europe. Glob. Ecol. Biogeogr..

[3] Lanello A., Kramer-Schadt S., Buchholz S., Gras P., von der Lippe M., Radchuk V. (2021). Arthropod Abundance Modulates Bird Community Responses to Urbanization. Divers. Distrib..

[4] Luniak M., Shaw W.W., Harris L.K., VanDruff L.V. (2004). Synurbization—Adaptation of Animal Wildlife to Urban Development. Proceedings of the 4th International Symposium on Urban Wildlife Conservation.

[5] Mikula P., Hromada M., Albrecht T., Tryjanowski P. (2014). Nest Site Selection and Breeding Success in Three Turdus Thrush Species Coexisting in an Urban Environment. Acta Ornithol..

[6] Lee J.-H., Kim S.-Y., Sung H.-C. (2024). Nest Site Selection, Nest Characteristics, and Breeding Ecology of the Eurasian Tree Sparrow, *Passer Montanus*, Living in an Urban Area. Anim. Taxon. Ecol..

[7] de Freitas R.M.P., Benvindo-Souza M., Sotero D.F., de Carvalho Lopes A.T., Santos M.A., de Oliveira J.M., da Costa D.C., Antoniosi Filho N.R., Vieira T.B., e Silva D.D.M. (2024). Ecotoxicological Consequences of Urbanization: A Multi-Biomarker Approach to Assessing Sewage Treatment Plant Effects on Free-Living Birds. Environ. Res..

[8] Xu X., Xie Y., Qi K., Luo Z., Wang X. (2018). Detecting the Response of Bird Communities and Biodiversity to Habitat Loss and Fragmentation Due to Urbanization. Sci. Total Environ..

[9] Djemadi I., Draidi K., Bakhouche B., Moussouni A., Bouslama Z. (2024). Recent Established Population of Tree Sparrow in Algeria: Palms Contributed Actively to Increasing Breeding Pairs. Ornithol. Res..

[10] Węgrzynowicz A. (2012). The Use of Nest-Boxes by Two Species of Sparrows (*Passer domesticus* and *P. montanus*) with Opposite Trends of Abundance—The Study in Warsaw. Int. Stud. Sparrows.

[11] Ramos-Elvira E., Banda E., Arizaga J., Martín D., Aguirre J.I. (2023). Long-Term Population Trends of House Sparrow and Eurasian Tree Sparrow in Spain. Birds.

[12] Cho D.-H., Lee J.-H., Lee M.-B., Oh S.-J., Sung H.-C. (2025). The Effect of Urbanisation on the Reproductive Performance and Development of Tree Sparrow *Passer montanus* Nestlings. Ardeola.

[13] Martínez-Miranzo B., López-García A., Payo-Payo A., Aguirre J.I., Banda E. (2026). Fitness Consequences of Urban Green Space Management in Eurasian Tree Sparrow (*Passer montanus*) in Madrid, Spain. Urban Sci..

[14] Seress G., Liker A. (2015). Habitat Urbanization and Its Effects on Birds. Acta Zool. Acad. Sci. Hung..

[15] Seress G., Hammer T., Bókony V., Vincze E., Preiszner B., Pipoly I., Sinkovics C., Evans K.L., Liker A. (2018). Impact of Urbanization on Abundance and Phenology of Caterpillars and Consequences for Breeding in an Insectivorous Bird. Ecol. Appl..

[16] Basile M., Augustinus B.A., Brockerhoff E.G. (2025). Tree Cover and Geographic Origin of Tree Species Drive Bird Functional Groups in Urban Environments. Wildl. Biol..

[17] Jokimäki J., Kaisanlahti-Jokimäki M.L. (2025). Nest Predation Pressure Differs Between Urban Ground- and Hole-Nesting Birds: Evidence from a Multi-Year Artificial Nest Predation Experiment. Birds.

[18] Chabani S., Khames G.E.Y., Djemadi I., Draidi K., Rezouani I.E., Mezhoud B., Moussouni A., Mederbal K.E., Telailia S., Bakhouche B. (2026). Breeding Under Pressure: Shorebird Reproductive Succes Amid Urban Disturbance Along a Mediterranean Urban Waterfront. Birds.

[19] Lindberg F., Grimmond C.S.B. (2011). Nature of Vegetation and Building Morphology Characteristics across a City: Influence on Shadow Patterns and Mean Radiant Temperatures in London. Urban Ecosyst..

[20] Javanroodi K., Nik V.M. (2019). Impacts of Microclimate Conditions on the Energy Performance of Buildings in Urban Areas. Buildings.

[21] Loss S.R., Will T., Loss S.S., Marra P.P. (2014). Bird–Building Collisions in the United States: Estimates of Annual Mortality and Species Vulnerability. Condor.

[22] Konstantopoulos K., Moustakas A., Vogiatzakis I.N. (2020). A Spatially Explicit Impact Assessment of Road Characteristics, Road-Induced Fragmentation and Noise on Birds Species in Cyprus. Biodiversity.

[23] Pinto C.M., Vargas Soto J.S., Flatt E., Barboza K., Whitworth A. (2024). Identifying Wildlife Road Crossing Mitigation Sites Using a Multi-Data Approach—A Case Study from Southwestern Costa Rica. J. Environ. Manag..

[24] Karlson M., Mörtberg U. (2015). A Spatial Ecological Assessment of Fragmentation and Disturbance Effects of the Swedish Road Network. Landsc. Urban Plan..

[25] Benítez-López A., Alkemade R., Verweij P.A. (2010). The Impacts of Roads and Other Infrastructure on Mammal and Bird Populations: A Meta-Analysis. Biol. Conserv..

[26] Fahrig L., Rytwinski T. (2009). Effects of Roads on Animal Abundance: An Empirical Review and Synthesis. Ecol. Soc..

[27] De Paiva Vianna K.M., Alves Cardoso M.R., Rodrigues R.M.C. (2015). Noise Pollution and Annoyance: An Urban Soundscapes Study. Noise Health.

[28] Montenegro A.L., Rey-Gozalo G., Arenas J.P., Suárez E. (2024). Streets Classification Models by Urban Features for Road Traffic Noise Estimation. Sci. Total Environ..

[29] Mulholland T.I., Ferraro D.M., Boland K.C., Ivey K.N., Le M.-L., Lariccia C.A., Vigianelli J.M., Francis C.D. (2018). Effects of Experimental Anthropogenic Noise Exposure on the Reproductive Success of Secondary Cavity Nesting Birds. Integr. Comp. Biol..

[30] Kang W., Minor E.S., Park C.R., Lee D. (2015). Effects of Habitat Structure, Human Disturbance, and Habitat Connectivity on Urban Forest Bird Communities. Urban Ecosyst..

[31] Miller J.R., Hobbs N.T. (2000). Recreational Trails, Human Activity, and Nest Predation in Lowland Riparian Areas. Landsc. Urban Plan..

[32] Strubbe D., Matthysen E. (2009). Experimental Evidence for Nest-Site Competition between Invasive Ring-Necked Parakeets (*Psittacula krameri)* and Native Nuthatches (*Sitta europaea*). Biol. Conserv..

[33] Lee J.-H., Nam W.-H., Sung H.-C. (2020). Breeding Ecology of the Eurasian Tree Sparrow *Passer Montanus* Living in Urban Area in South Korea. Korean J. Ornithol..

[34] Šálek M., Riegert J., Grill S. (2015). House Sparrows *Passer domesticus* and Tree Sparrows *Passer montanus*: Fine-Scale Distribution, Population Densities, and Habitat Selection in a Central European City. Acta Ornithol..

[35] McHugh N.M., Prior M., Grice P.V., Leather S.R., Holland J.M. (2017). Agri-Environmental Measures and the Breeding Ecology of a Declining Farmland Bird. Biol. Conserv..

[36] Havlíček J., Riegert J., Bandhauerová J., Fuchs R., Šálek M. (2021). Species-Specific Breeding Habitat Association of Declining Farmland Birds within Urban Environments: Conservation Implications. Urban Ecosyst..

[37] Lee D.H., Eom T.K., Lee J.K., Ko H., Kim J.H., Ahn S.H., Rhim S.J. (2025). Effects of Ecological Factors on Occupancy of Artificial Nest Boxes by the Great Tit (*Parus major*): A Comprehensive Analysis. Pak. J. Zool..

[38] Dulisz B., Stawicka A.M., Knozowski P., Diserens T.A., Nowakowski J.J. (2022). Effectiveness of Using Nest Boxes as a Form of Bird Protection after Building Modernization. Biodivers. Conserv..

[39] Arizaga J., Iraeta A., Crespo A., Azkona A., Banda E., Laso M., Gutiérrez Ó. (2023). Programas de Monitorización de Aves a Largo Plazo de la Oficina de Anillamiento de la Sociedad de Ciencias Aranzadi.

[40] Field R.H., Anderson G.Q.A. (2004). Habitat Use by Breeding Tree Sparrows *Passer montanus*. IBIS.

[41] QGIS Development Team (2024). QGIS Geographic Information System.

[42] Shepard D. (1968). A Two-Dimensional Interpolation Function for Irregularly Spaced Data. Proceedings of the 1968 23rd ACM National Conference.

[43] Akaike H. (1973). Information Theory and an Extension of the Maximum Likelihood Principle. Proceedings of the 2nd International Symposium on Information Theory.

[44] R Core Team (2024). R: A Language and Environment for Statistical Computing.

[45] Chamberlain D.E., Cannon A.R., Toms M.P., Leech D.I., Hatchwell B.J., Gaston K.J. (2009). Avian Productivity in Urban Landscapes: A Review and Meta-Analysis. IBIS.

[46] Seress G., Bókony V., Pipoly I., Szép T., Nagy K., Liker A. (2012). Urbanization, Nestling Growth and Reproductive Success in a Moderately Declining House Sparrow Population. J. Avian Biol..

[47] Chang H.Y., Lee Y.F. (2016). Effects of Area Size, Heterogeneity, Isolation, and Disturbances on Urban Park Avifauna in a Highly Populated Tropical City. Urban Ecosyst..

[48] Bodnaruk E.W., Kroll C.N., Yang Y., Hirabayashi S., Nowak D.J., Endreny T.A. (2017). Where to Plant Urban Trees? A Spatially Explicit Methodology to Explore Ecosystem Service Tradeoffs. Landsc. Urban Plan..

[49] Cimburova Z., Berghauser Pont M. (2021). Location Matters. A Systematic Review of Spatial Contextual Factors Mediating Ecosystem Services of Urban Trees. Ecosyst. Serv..

[50] Logan J.D., Wolesensky W., Joern A. (2006). Temperature-Dependent Phenology and Predation in Arthropod Systems. Ecol. Modell..

[51] Salman I.N.A., Blaustein L. (2018). Vegetation Cover Drives Arthropod Communities in Mediterranean/Subtropical Green Roof Habitats. Sustainability.

[52] Tan P.Y., Ismail M.R. (2015). Bin The Effects of Urban Forms on Photosynthetically Active Radiation and Urban Greenery in a Compact City. Urban Ecosyst..

[53] Stauss M.J., Burkhardt J.F., Tomiuk J. (2005). Foraging Flight Distances as a Measure of Parental Effort in Blue Tits Parus Caeruleus Differ with Environmental Conditions. J. Avian Biol..

[54] Low M., Arlt D., Eggers S., Pärt T. (2010). Habitat-Specific Differences in Adult Survival Rates and Its Links to Parental Workload and on-Nest Predation. J. Anim. Ecol..

[55] Evens R., Beenaerts N., Neyens T., Witters N., Smeets K., Artois T. (2018). Proximity of Breeding and Foraging Areas Affects Foraging Effort of a Crepuscular, Insectivorous Bird. Sci. Rep..

[56] Schroeder J., Nakagawa S., Cleasby I.R., Burke T. (2012). Passerine Birds Breeding under Chronic Noise Experience Reduced Fitness. PLoS ONE.

[57] Francis C.D., Ortega C.P., Cruz A. (2009). Noise Pollution Changes Avian Communities and Species Interactions. Curr. Biol..

[58] Zhou B., Liu J., Liang W. (2020). Breeding in a Noisy World: Attraction to Urban Arterial Roads and Preference for Nest-Sites by the Scaly-Breasted Munia (*Lonchura punctulata*). Glob. Ecol. Conserv..

[59] Fernández-Juricic E., Jokimäki J. (2001). A Habitat Island Approach to Conserving Birds in Urban Landscapes: Case Studies from Southern and Northern Europe. Biodivers. Conserv..

[60] Remacha C., Delgado J.A. (2009). Spatial Nest-Box Selection of Cavity-Nesting Bird Species in Response to Proximity to Recreational Infrastructures. Landsc. Urban Plan..

