# Supplementary material for: Breeding in the City: Non-Linear Associations of Urbanization, Tree Cover, and Human Disturbance with Eurasian Tree Sparrows (Passer montanus) Breeding Performance
Source: Biology (Basel). 2026 Oct 2;15(19):1746. doi: 10.3390/biology15191746 (PMC13663493; doi:10.3390/biology15191746)
Supplement: Supplementary file 1 [file biology-15-01746-s001.zip › Supplementary material.pdf]

SUPPLEMENTARY MATERIALS:

The first principal component (PC1) of the principal component analysis was interpreted as an urbanization gradient, explaining 35.8% of the total variance. The second principal component (PC2) accounted for an additional 27.1% of the variance (Figure. S1; Table A1). However, PC2 (eigenvalue = 1.6279) was not retained, as the objective was to derive a single urbanization index, and this component represented a pattern not relevant to the aims of the study.

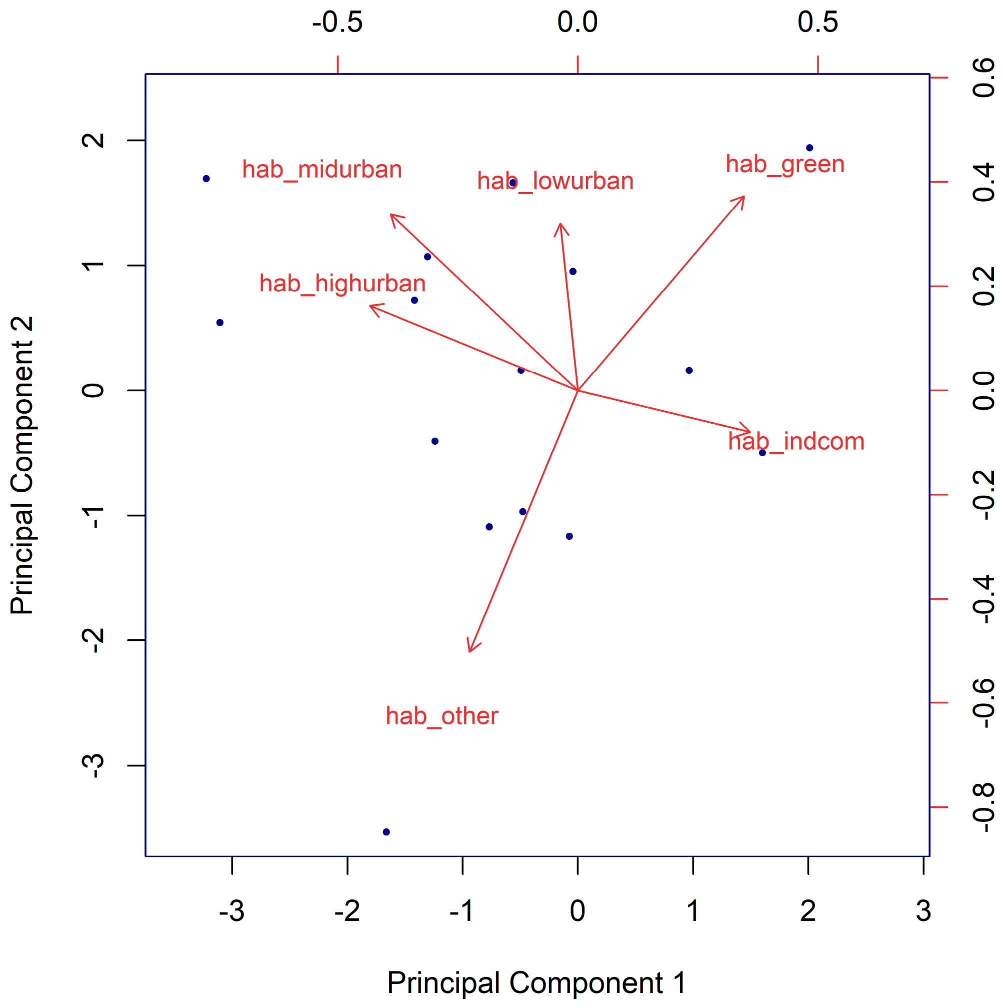

Figure S1. Principal component scores and loadings. The axes represent the first two principal components, and the arrows indicate the contribution and direction of each variable. *hab_highurban* denotes habitats with high urban density, *hab_midurban* medium-density urban areas, *hab_lowurban* low-density urban areas, *hab_other* other habitat types, *hab_green*, green areas, and *hab_indcom* commercial and industrial areas.

Table S1. Variance explained and loadings of the principal components describing the degree of urbanization.

	Comp.1	Comp.2	Comp.3	Comp.4	Comp.5	Comp.6
**Standard deviation**	1.4664	1.2759	1.1100	0.8401	0.5328	5.5755 e-08
**Proportion of Variance**	0.3584	0.2713	0.2054	0.1176	0.0473	5.1810 e-16
**Cumulative Proportion**	0.3584	0.6297	0.8350	0.9527	1.0000	1.0000

In this case, PC1 was not retained as it did not capture any pattern of interest. In contrast, PC2 (eigenvalue = 1.1665), explaining 23.3% of the total variance, was interpreted as a gradient of road intensity associated with traffic speed (Figure. S2; Table S2).

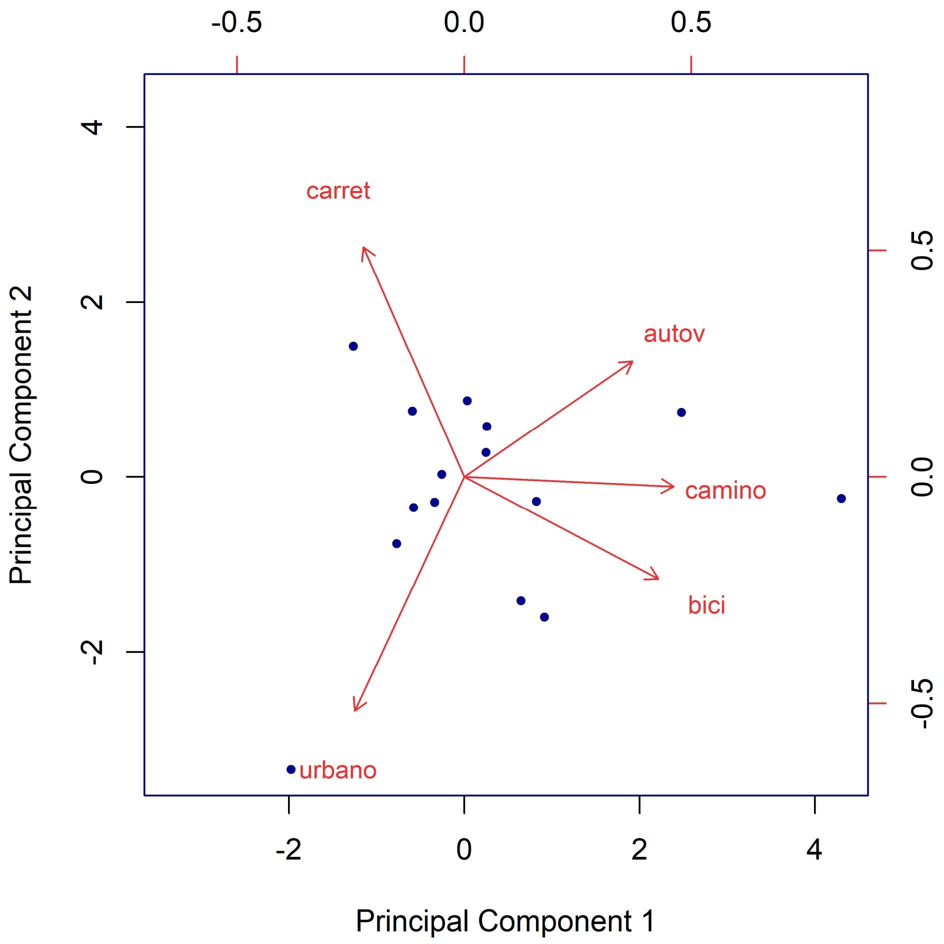

Figure S2. Principal component scores and loadings. The axes represent the first two principal components, and the arrows indicate the contribution and direction of each variable. *autov* denotes motorways and highways, *camino* paths and tracks, *carret* conventional roads, *bici* bike lanes, and *urbano* urban roads.

Table S2. Variance explained and loadings of the principal components describing road-related variables.

	Comp.1	Comp.2	Comp.3	Comp.4	Comp.5
**Standard deviation**	1.4168453	1.0800393	0.8538349	0.8111655	0.6626017
**Proportion of Variance**	0.4014901	0.2332970	0.1458068	0.1315979	0.0878082
**Cumulative Proportion**	0.4014901	0.6347871	0.7805939	0.9121918	1.0000

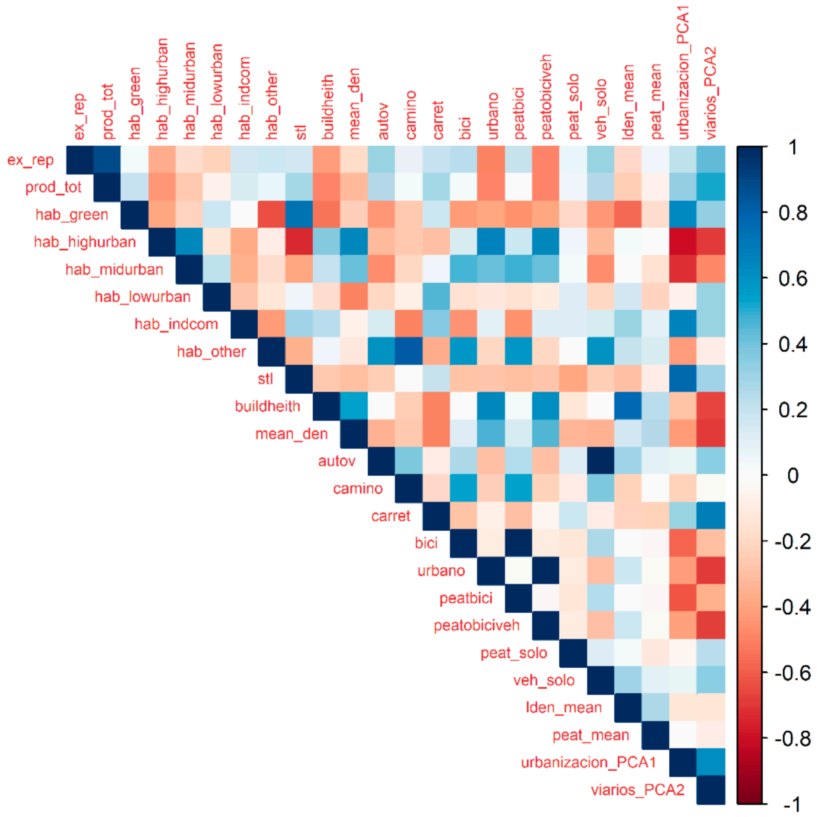

Figure S3. Correlation matrix of the urban variables included in the study. Colors indicate the direction and magnitude of correlations (red: negative; blue: positive), and color intensity reflects the strength of the relationship, with values ranging from -1 to +1.

Table S3. Variance inflation factors (VIF), generalized variance inflation factors (GVIF), and adjusted GVIF values for predictors included in the final models.

Model	Predictor	df	VIF/GVIF	Adjusted GVIF
Occupancy	urb_cat	3	1.696	1.092
	peat_mean	1	1.040	1.020
	buildheith	1	1.732	1.316
Reproductive succes	cajas_dens	1	0.281	0.530
	peat_mean	1	0.300	0.548
	lden_mean	1	1.860	1.364
	buildheith	1	1.758	1.326
	stl	1	0.281	0.530
Average clutches	urb_cat	3	9.449	1.454
	cajas_dens	1	1.785	1.336
	lden_mean	1	5.570	2.360
	buildheith	1	7.913	2.813
	vial_cat	3	18.075	1.620
Total eggs per nest boxes	ano	12	11.469	1.107
	urb_cat	3	17.094	1.605
	cajas_dens	1	2.256	1.502
	peat_mean	1	4.198	2.049
	buildheith	1	3.527	1.878
	vial_cat	3	14.357	1.559
Total chicks per nest boxes	ano	12	11.469	1.107
	urb_cat	3	17.094	1.605
	cajas_dens	1	2.256	1.502
	peat_mean	1	4.198	2.049
	buildheith	1	3.527	1.878
	vial_cat	3	14.357	1.559
Total nestlings reaching ringing age per nest boxes	urb_cat	3	7.089	1.386
	peat_mean	1	1.166	1.080
	lden_mean	1	1.243	1.115
	vial_cat	3	6.877	1.379

Note: *VIF and GVIF values are reported according to the degrees of freedom of each predictor. Adjusted GVIF values [GVIF^(1/(2 × Df))] are reported for all predictors to facilitate comparison among variables with different degrees of freedom. The productivity model including the urbanization × tree cover interaction (GLM3) is not included because the interaction term for the partially urbanized category was non-estimable due to a lack of variation in tree cover within that category.*

Table S4. Full coefficients for the final models.

Response variable	Predictor	β	SE	t	p
**Occupancy**	***Interecept***	3.171	0.714	4.444	**<0.001**
	urb_cat2	1.692	0.300	5.641	**<0.001**
	urb_cat3	-0.067	0.282	-0.236	0.814
	urb_cat4	-0.561	0.318	-1.763	0.084
	peat_mean	-0.0013	0.0005	-2.615	**0.012**
	buildheith	-0.277	0.052	-5.372	**<0.001**
**Total productivity**	***Intercept***	14.748	4.700	3.138	**0.003**
	urb_cat2	1.421	0.855	1.663	0.102
	urb_cat3	-3.343	3.644	-0.917	0.363
	urb_cat4	-9.103	3.060	-2.975	**0.004**
	stl	-13.333	8.701	-1.532	0.132
	buildheith	-0.357	0.112	-3.203	**0.002**
	urb_cat3*stl	1.017	9.251	0.110	0.913
	urb_cat4*stl	29.230	8.985	3.253	**0.002**
**Reproductive succes**	***Intercept***	-0.975	1.899	-0.514	0.610
	cajas_dens	0.007	0.007	1.011	0.317
	peat_mean	0.001	0.001	0.755	0.454
	Lden_mean	0.058	0.040	1.442	0.155
	buildheith	-0.209	0.067	-3.127	**0.003**
	stl	0.760	1.006	0.755	0.453
**Average clutches**	***Intercept***	0.168	0.915	0.184	0.855
	urb_cat2	1.305	0.285	4.584	**<0.001**
	urb_cat3	-0.164	0.216	-0.756	0.453
	urb_cat4	0.118	0.266	0.442	0.660
	cajas_dens	0.006	0.003	1.758	0.085
	lden_mean	0.062	0.021	2.899	**0.006**
	buildheith	-0.189	0.042	-4.515	**<0.001**
	vial_cat2	0.212	0.272	0.780	0.439
	vial_cat3	-0.782	0.290	-2.693	**0.010**
	vial_cat4	0.056	0.447	0.125	0.901
**Total eggs per nest boxes**	***Intercept***	3,675	1,315	2,794	**0,008**
	ano2013	1,716	1,168	1,469	0,150
	ano2014	0,922	1,168	0,789	0,435
	ano2015	1,763	1,168	1,509	0,139
	ano2016	0,864	1,168	0,740	0,464
	ano2017	1,923	1,168	1,646	0,108
	ano2018	2,650	1,168	2,268	0,029
	ano2019	1,750	1,017	1,721	0,093
	ano2020	1,845	0,994	1,856	0,071
	ano2021	2,462	1,030	2,389	**0,022**
	ano2022	6,003	1,327	4,523	**<0.001**
	ano2023	5,150	1,220	4,221	**<0.001**
	ano2024	5,311	1,180	4,502	**<0.001**
	urb_cat2	3,249	0,816	3,979	**<0.001**
	urb_cat3	-0,044	0,700	-0,063	0,950
	urb_cat4	-0,927	0,862	-1,076	0,289
	cajas_dens	0,013	0,011	1,192	0,241
	peat_mean	-0,009	0,001	-6,082	**<0.001**
	buildheith	-0,088	0,076	-1,152	0,257
	vial_cat2	-0,250	0,768	-0,326	0,747
	vial_cat3	-1,542	0,805	-1,915	0,063
	vial_cat4	-0,522	1,015	-0,514	0,610

**Total chicks per nest boxes**	***Intercept***	2,269	1,129	2,009	0,052
	ano2013	1,358	1,003	1,354	0,184
	ano2014	1,410	1,003	1,405	0,168
	ano2015	1,962	1,003	1,956	0,058
	ano2016	1,287	1,003	1,283	0,207
	ano2017	1,999	1,003	1,993	0,054
	ano2018	2,255	1,003	2,248	**0,030**
	ano2019	2,062	0,873	2,361	**0,023**
	ano2020	2,192	0,853	2,568	**0,014**
	ano2021	2,686	0,885	3,036	0,004
	ano2022	5,260	1,140	4,616	**<0.001**
	ano2023	4,340	1,048	4,143	**<0.001**
	ano2024	4,278	1,013	4,223	**<0.001**
	urb_cat2	2,390	0,701	3,409	**0,002**
	urb_cat3	-0,130	0,601	-0,216	0,830
	urb_cat4	-0,985	0,740	-1,331	0,191
	cajas_dens	0,011	0,009	1,227	0,228
	peat_mean	-0,007	0,001	-5,120	**<0.001**
	buildheith	-0,091	0,065	-1,391	0,172
	vial_cat2	0,025	0,660	0,039	0,969
	vial_cat3	-0,993	0,691	-1,436	0,159
	vial_cat4	-0,064	0,871	-0,074	0,942
**Total nestlings reaching ringing age per nest boxes**	***Intercept***	10,677	1,302	8,202	**<0.001**
	urb_cat2	2,506	0,602	4,161	**<0.001**
	urb_cat3	0,728	0,445	1,634	0,108
	urb_cat4	0,179	0,543	0,329	0,744
	peat_mean	-0,001	0,001	-2,124	0,039
	lden_mean	-0,137	0,022	-6,157	**<0.001**
	vial_cat2	-0,767	0,531	-1,444	0,155
	vial_cat3	-1,825	0,551	-3,314	**0,002**
	vial_cat4	-2,008	0,696	-2,884	**0,006**
